# The potential of hyperpolarised ^13^C-MRI to target glycolytic tumour core in prostate cancer

**DOI:** 10.1007/s00330-022-08929-7

**Published:** 2022-06-22

**Authors:** Nikita Sushentsev, Mary A. McLean, Anne Y. Warren, Cara Brodie, Julia Jones, Ferdia A. Gallagher, Tristan Barrett

**Affiliations:** 1 Department of Radiology, Addenbrooke’s Hospital and University of Cambridge School of Clinical Medicine, Box 218, Cambridge Biomedical Campus, Cambridge, CB2 0QQ UK; 2 Cancer Research UK Cambridge Institute, University of Cambridge, Cambridge, UK; 3 Department of Pathology, Cambridge University Hospitals NHS Foundation Trust, Cambridge, UK

**Keywords:** Prostatic neoplasms, Molecular imaging, Magnetic resonance imaging

## Abstract

**Abstract:**

Hyperpolarised [1-^13^C]pyruvate MRI (HP-^13^C-MRI) is an emerging metabolic imaging technique that has shown promise for evaluating prostate cancer (PCa) aggressiveness. Accurate tumour delineation on HP-^13^C-MRI is vital for quantitative assessment of the underlying tissue metabolism. However, there is no consensus on the optimum method for segmenting HP-^13^C-MRI, and whole-mount pathology (WMP) as the histopathological gold-standard is only available for surgical patients. Although proton MRI can be used for tumour delineation, this approach significantly underestimates tumour volume, and metabolic tumour segmentation based on HP-^13^C-MRI could provide an important functional metric of tumour volume. In this study, we quantified metabolism using HP-^13^C-MRI and segmentation approaches based on WMP maps, ^1^H-MRI-derived T_2_-weighted imaging (T2WI), and HP-^13^C-MRI-derived total carbon signal-to-noise ratio maps (TC-SNR) with an SNR threshold of 5.0. ^13^C-labelled pyruvate SNR, lactate SNR, TC-SNR, and the pyruvate-to-lactate exchange rate constant (*k*_PL_) were significantly higher when measured using the TC-SNR-guided approach, which also corresponded to a significantly higher tumour epithelial expression on RNAscope imaging of the enzyme catalysing pyruvate-to-lactate metabolism (lactate dehydrogenase (LDH)). However, linear regression and Bland-Altman analyses demonstrated a strong linear relationship between all three segmentation approaches, which correlated significantly with RNA-scope-derived epithelial LDH expression. These results suggest that standard-of-care T2WI and TC-SNR maps could be used as clinical reference tools for segmenting localised PCa on HP-^13^C-MRI in the absence of the WMP gold standard. The TC-SNR-guided approach could be used clinically to target biopsies towards highly glycolytic tumour areas and therefore to sample aggressive disease with higher precision.

**Key Points:**

*• T2WI- and TC-SNR-guided segmentations can be used in all PCa patients and do not explicitly require WMP maps.*

*• Agreement between the three segmentation approaches is biologically validated by their strong relationship with epithelial LDH mRNA expression.*

• *The TC-SNR-guided approach can potentially be used to identify occult disease on* ^*1*^*H-MRI and target the most glycolytically active regions.*

**Supplementary Information:**

The online version contains supplementary material available at 10.1007/s00330-022-08929-7.

## Introduction

Hyperpolarised [1-^13^C]pyruvate MRI (HP-^13^C-MRI) is a rapidly evolving metabolic imaging technique that can visualise real-time pyruvate-to-lactate conversion, catalysed by the enzyme lactate dehydrogenase (LDH) [1]. Several studies have shown increased [1-^13^C]lactate labelling in localised prostate cancer (PCa), highlighting the added value of HP-^13^C-MRI to standard-of-care imaging for assessing tumour aggressiveness and monitoring treatment response [1–4]. However, despite the promise shown by these early studies, there remain several technical challenges that require optimisation to ensure that HP-^13^C-MRI assessment is reliable and reproducible prior to clinical translation of the technique.

In localised disease, accurate tumour delineation is vital for definitive evaluation of the underlying tissue metabolism. However, whole-mount pathology (WMP) is only available following radical prostatectomy, making the histopathological gold standard for tumour delineation inaccessible for most patients in which HP-^13^C-MRI may be of clinical benefit. Conventional proton (^1^H) MRI is known to underestimate tumour diameter and volume up by up to 3-fold when compared to WMP [5], and may therefore under-sample metabolically active areas if used for lesion segmentation. The relationship between the area of high [1-^13^C]lactate signal and the underlying tumour area on pathology is unknown, and could vary from representing only the metabolically active tumour core, through detecting labelled lactate beyond the anatomical boundaries of the lesion. In the former case, direct lesion segmentation on HP-^13^C-MRI may be sufficient for accurate tumour metabolic phenotyping based on assessing the glycolytic volume, which is known to harbour more aggressive disease [2, 4, 6]. This is analogous to approaches used in positron emission tomography (PET) to delineate the metabolically active tumour volume based on thresholding the ^18^F-fluorodeoxyglucose (^18^F-FDG) uptake [7].

This study aimed to identify a clinically applicable HP-^13^C-MRI segmentation approach that would best reflect underlying tumour metabolic characteristics. Tumour HP-^13^C-MRI-derived metabolic metrics and spatial LDH mRNA expression patterns were compared using three segmentation approaches: WMP, ^1^H-MRI, and HP-^13^C-MRI-derived total carbon signal-to-noise ratio maps (TC-SNR).

## Materials and methods

This was a retrospective study of data acquired prospectively as part of an institutional review board–approved (National Research Ethics Service Committee East of England, Cambridge South, 16/EE/0205) study that included patients undergoing radical prostatectomy for biopsy-proven PCa [4]. All patients underwent 3 T pre-biopsy mpMRI and HP-^13^C-MRI, measuring pyruvate SNR, lactate SNR, TC-SNR, and the pyruvate-to-lactate exchange rate constant (*k*_PL_). Full imaging protocols for mpMRI and HP-^13^C-MRI have been described previously [4]. All patients provided written consent to participate in this study upon their recruitment between May 2018 and February 2020, with the reported data analysis covered by the initial institutional review board approval.

### Image segmentation and analysis

**Fig. 1:**
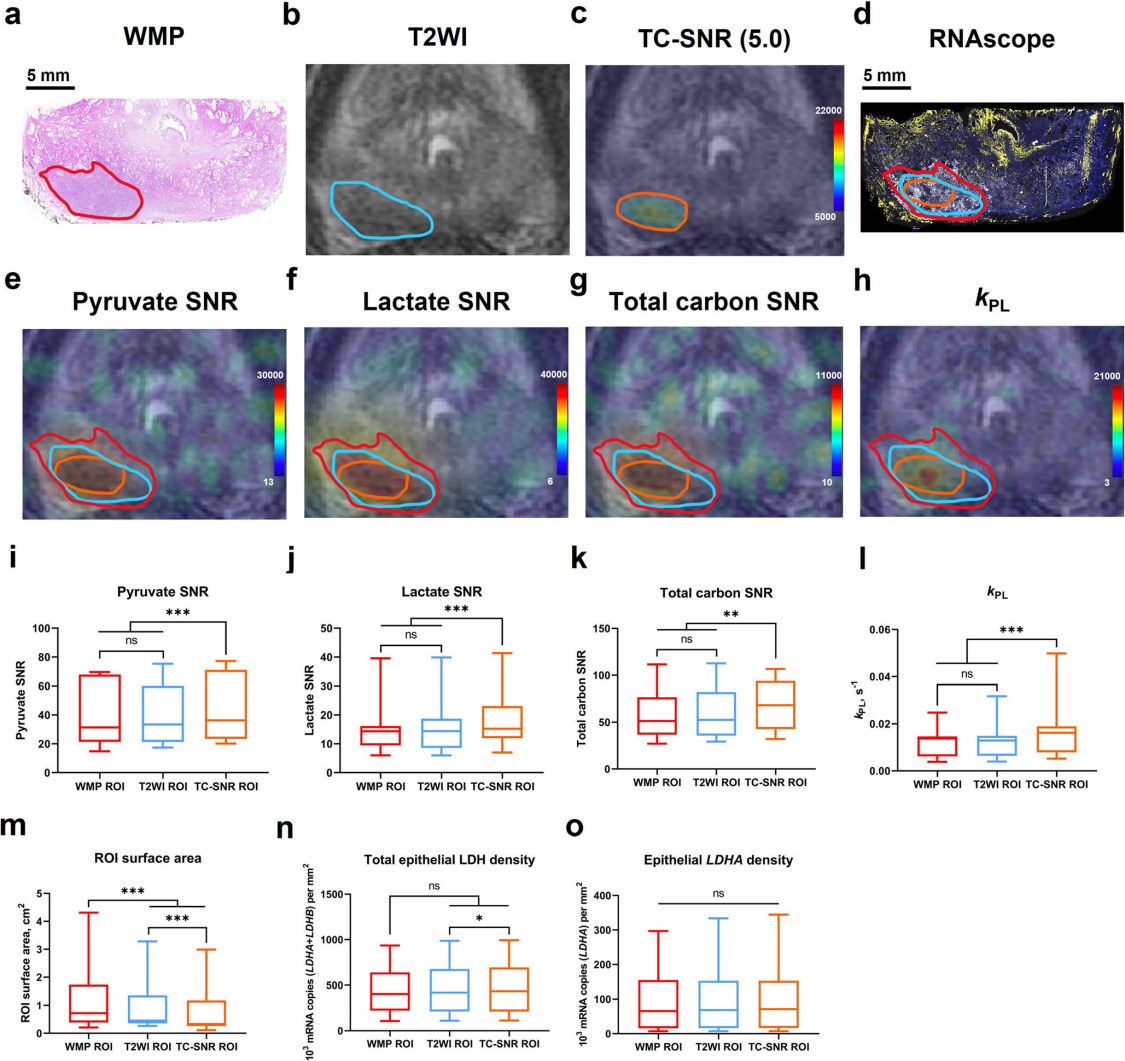
WMP map (**a**), ^1^H-MRI-derived T2WI (**b)**, HP ^13^C-MRI-derived TC-SNR map with the lowest SNR threshold set at 5.0 (**c**), RNAscope WMP fluorescent image showing *LDHA* (gold) and *LDHB* (white) expression (**d**). WMP- (**a**), T2WI- (**b**), and TC-SNR-guided (**c**) tumour segmentations, with all three resulting ROIs overlaid on the RNAscope image (**d**). HP ^13^C-MRI-derived pyruvate SNR (**e**), lactate SNR (**f**), total carbon SNR (**g**), and *k*_PL_ (**h**) maps with WMP-, T2WI-, and TC-SNR-guided segmentations overlaid. Panels **a–h** were obtained from a 66-year-old patient with a histopathologically confirmed Gleason score 3 + 4 = 7 peripheral zone lesion (red ROI on panel **a**). Box-and-whisker plots comparing HP ^13^C-MRI-derived pyruvate SNR (**i**), lactate SNR (**j**), TC-SNR (**k**), and *k*_PL_ (**l**), alongside ROI surface areas (**m**), RNAscope-derived total epithelial LDH density (**n**), and epithelial *LDHA* density measured using the WMP-, T2WI-, and TC-SNR-guided segmentation approaches

PCa foci were first outlined on digitised surgical haematoxylin-and-eosin (H&E) WMP maps by an experienced genitourinary pathologist (A.Y.W.) (WMP-guided segmentation; Fig. 1a) using XPlore (Koninklijke Philips). Corresponding lesions were independently outlined on mpMRI-derived T_2_-weighted images (T2WI) in consensus by two radiologists (T.B., F.A.G.) and a research fellow (N.S.) with 12, 13, and 4 years’ experience in prostate MRI, respectively. The segmentation was blinded to WMP maps, with PCa regions-of-interest (ROIs) defined by clinical interpretation as areas showing low T2WI signal intensity with corresponding restriction on diffusion-weighted imaging according to clinical guidelines [8], and concordant to targeted biopsy results (T2WI-guided segmentation, Fig. 1b). Separately, the same readers re-segmented the lesions on HP-^13^C-MRI-derived TC-SNR maps, masked using a TC-SNR threshold of 5.0 according to the Rose criterion [9] to remove all pixels potentially containing noisy data (TC-SNR-guided segmentation, Figure 1c). At the end of each segmentation session, the resulting ROIs were transposed onto co-registered HP-^13^C-MRI metabolic maps (Fig. 1e–h), from which pyruvate SNR, lactate SNR, TCSNR, and *k*_PL_ values were extracted for each pixel using OsiriX 10.0 (Pixmeo SARL).

### Lactate dehydrogenase mRNA expression analysis

Simultaneous detection of human *LDHA* and *LDHB* was performed in 4-μm-thick whole-mount formalin-fixed, paraffin-embedded (FFPE) prostatectomy tumour blocks using RNAscope in situ hybridisation technology, as detailed previously [4]. The slides were analysed using HALO v3.2.1851.266 (Indica Labs) and the FISH v2.2.0 module. Epithelial and stromal cell populations were differentiated using a fluorescent random forest tissue classifier [4]. Epithelial *LDHA* and *LDHB* mRNA expression values were derived from WMP-, T2WI-, and TC-SNR-guided segmentations that were transposed onto co-registered RNAscope slides (Figure 1d). To account for the differences in the ROI surface areas, the combined epithelial LDH expression (sum of *LDHA* and *LDHB* mRNA copies per ROI) and epithelial *LDHA* expression values were divided by the ROI surface area (mm^2^) to produce the total epithelial LDH and epithelial *LDHA* densities, respectively.

### Statistical analysis

Statistical analyses were conducted using GraphPad Prism (version 9.1.2, GraphPad Software). Data normality was assessed using the D’Agostino-Pearson test, with all pairwise comparisons conducted using the Wilcoxon matched-pairs signed-rank test. Spatial congruence between different segmentation approaches was assessed using the Dice similarity coefficient (DSC) [10]. Simple linear regression was used to evaluate the relationship between HP-13C-MRI values derived using these approaches, and agreement was assessed by Bland-Altman analysis. For the latter, whilst some mean differences between measurements did not follow normal distribution (Supplementary Information), we did not perform their logarithmic transformation since in all cases there was no relationship between the magnitude of the quantity being measured (mean differences) and the difference between them, and 95% of non-transformed observations were already included in the limits of agreement (LoA), thereby meeting the key assumption behind the use of the LoA approach. Spearman’s rank correlation test assessed relationships between HP-^13^C-MRI-derived lactate SNR and *k*_PL_, and RNAscope-derived total epithelial LDH density and epithelial *LDHA* density, respectively, for the three segmentation approaches.

## Results

The study comprised eight patients (57–69 years; PSA 3.1–19.1 ng/mL) with 11 MR-visible lesions, nine and two of which harboured Likert scores 5 and 4, respectively. Seven lesions were in the peripheral and four in the transition zones, with the median maximum axial tumour diameter measuring 16 mm (interquartile range, 12–21 mm). One tumour harboured Gleason score 3 + 3 = 6 disease, eight 3 + 4 = 7 disease, and two 4 + 3 = 7 disease.

Pyruvate SNR, lactate SNR, TC-SNR, and *k*_PL_ showed no significant differences between the WMP- and T2WI-guided segmentation approaches (*p* > 0.05; Fig. 1i–l, Table 1), which is explained by their high spatial congruence (DSC = 0.86), despite WMP-guided ROIs having a significantly larger median surface area (*p* = 0.005; Fig. 1m, Table 1). Conversely, all TC-SNR-guided metabolic metrics were significantly higher compared to those obtained using the two other approaches (*p* < 0.05 for all; Fig. 1i–l, Table 1), with TCSNR-guided ROIs also being significantly smaller compared to both WMP- and T2WI-guided ROIs (*p* = 0.001 for both; Fig. 1m, Table 1). However, TC-SNR-guided ROIs showed high spatial congruence with both WMP- and T2WI-guided ROIs as demonstrated by the DSCs of 0.85 and 0.86, respectively. Importantly, total epithelial LDH density was significantly higher when measured using the TC-SNR-guided approach compared to the T2WI-based segmentation (*p* = 0.02; Fig. 1n, Table 1), whilst no difference was observed when comparing both T2WI- and TC-SNR-guided values with the WMP-guided approach (*p* = 0.13 and 0.08, respectively; Fig. 1n, Table 1).

**Table 1:** Comparison of HP ^13^C-MRI metabolic parameters, ROI surface areas, and RNA scope–derived total epithelial LDH density and epithelial *LDHA* values measured using different segmentation approaches. The data are presented as median (interquartile range). *k*_PL_ values are presented as s^−1^, ROI surface areas are presented as cm^2^, and total epithelial LDH density values are presented as (*LDHA* + *LDHB* mRNA copies) per mm^2^. *p* values were obtained using the Wilcoxon matched-pairs signed-rank test given the outcomes of the D’Agostino-Pearson test used to assess the data normality

Parameter	WMP-guided segmentation	T2WI-guided segmentation	TC-SNR-guided segmentation	*p* value, 1 vs 2	*p* value, 1 vs 3	*p* value, 2 vs 3
Pyruvate SNR	31.4 (21.4–67.9)	33.5 (21.3–60.2)	36.3 (22.6–61.2)	0.831	0.001*	0.001
Lactate SNR	14.4 (9.5–16.2)	12.4 (8.6–18.7)	15.2 (9.2–23.1)	0.700	0.001*	0.001*
Total carbon SNR	51.3 (36.7–76.8)	52.5 (35.7–82.2)	68.2 (42.7–94.1)	0.240	0.003*	0.007*
*k*_PL_	0.011 (0.005–0.015)	0.010 (0.008–0.018)	0.012 (0.005–0.018)	0.206	0.001*	0.001*
ROI surface area	0.72 (0.39–1.74)	0.46 (0.36–1.36)	0.36 (0.32–1.18)	0.005*	0.001*	0.001*
Total epithelial LDH density	404.2 (222.1–639.5)	419.8 (211.6–677.4)	434.9 (212.1–693.8)	0.134	0.081	0.020*
Epithelial *LDHA* density	65.6 (15.9–155.0)	68.6 (16.1–152.9)	71.1 (16.5–152.9)	0.426	0.203	0.098

*p < 0.05

*CI* confidence interval, *LDH* lactate dehydrogenase, *ROI* region-of-interest, *SNR* signal-to-noise ratio, *T2WI* T_2_-weighted imaging, *WMP* whole-mount pathology

**Fig. 2:**
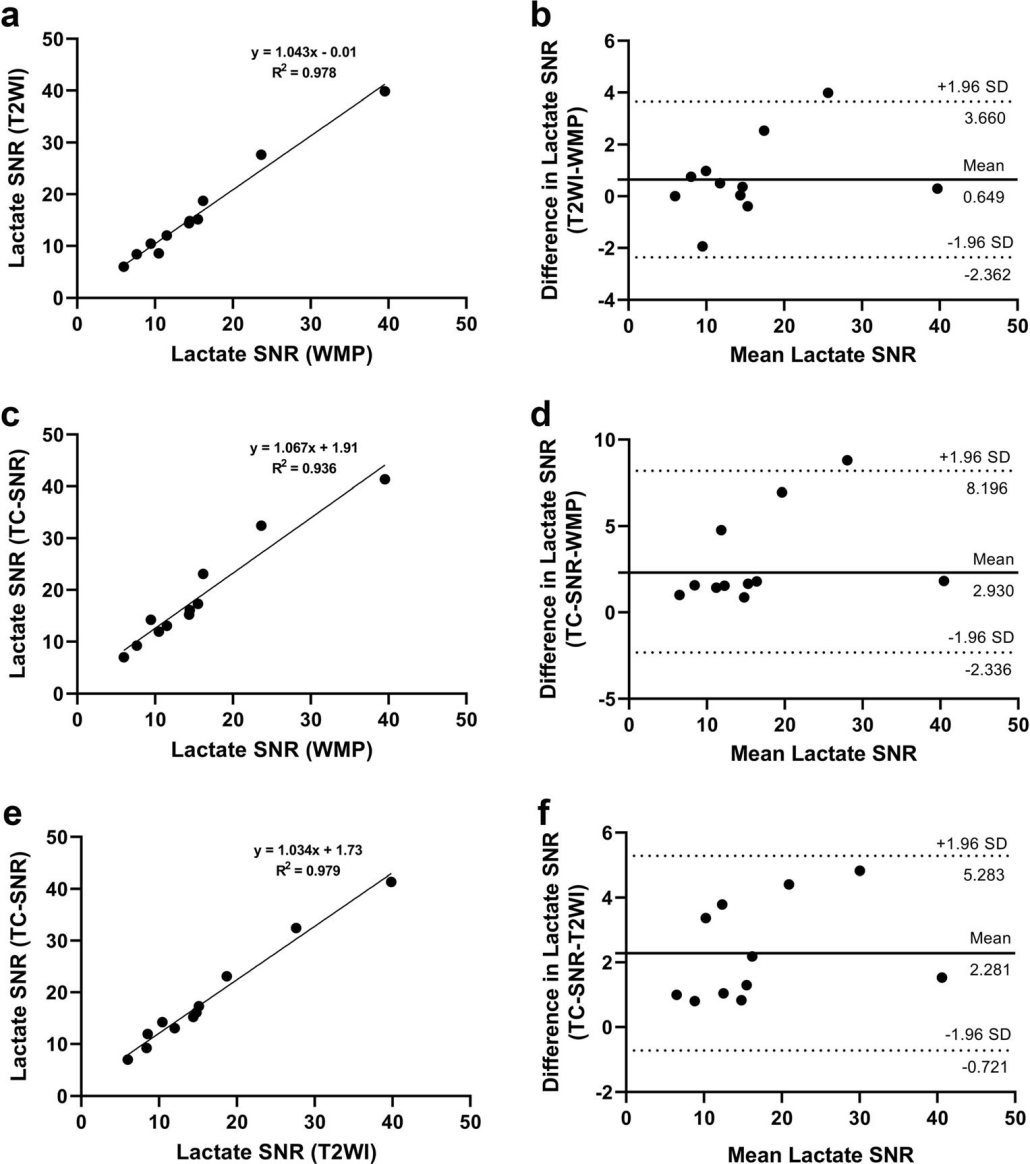
Linear regression plots (**a**, **c**, **e**) and Bland-Altman plots (**b**, **d**, **f**) comparing HP ^13^C-MRI-derived lactate SNR values obtained using WMP-, T2WI-, and TC-SNR-guided segmentation approaches. **a**, **c**, **e** Linear regression plots include captions representing slopes of the linear fits, *y*-intercepts, and coefficients of determination (*R*^2^). **b**, **d**, **f** Bland-Altman plots include dotted lines representing upper and lower 95% limits of agreement and bold lines representing the mean biases with appropriate captions included. Similar plots for pyruvate SNR, TC-SNR, and *k*_PL_ are presented in the Supplementary Information

Linear regression analysis showed very strong positive correlations between lactate SNR from all three segmentation approaches (Fig. 2a, c, e). Bland-Altman analysis showed excellent agreement among the three segmentation methods as exemplified by lactate SNR measurements (Fig. 2b, d, f), with the best agreement noted between TC-SNR- and T2WI-guided segmentation approaches, whereby all individual lactate SNR values were within the 95% limits of agreement (Fig. 2f). The outcomes of both linear regression and Bland-Altman analyses were similar for other HP-^13^C-MRI-derived metrics (Supplementary Information).

**Fig. 3:**
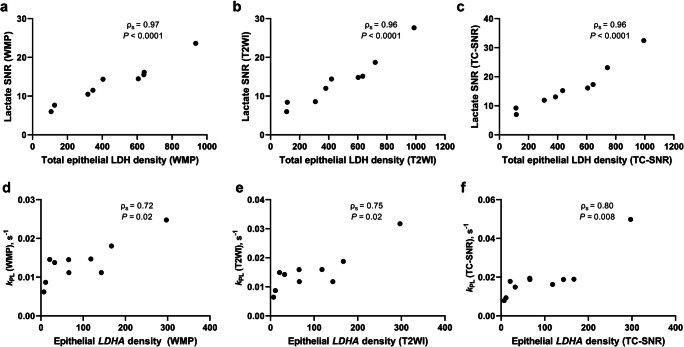
Spearman’s correlation plots comparing RNAscope-derived total epithelial LDH values with HP ^13^C-MRI-derived lactate SNR values obtained using WMP- (**a**), T2WI (**b**), and TC-SNR-guided (**c**) segmentation approaches. Spearman’s correlation plots comparing RNAscope-derived epithelial *LDHA* density values with HP ^13^C-MRI-derived *k*_PL_ values obtained using WMP- (**d**), T2WI- (**e**), and TC-SNR-guided (**f**) segmentation approaches. Plots **a–f** include individual rank correlation coefficients and corresponding *p* values

Finally, Spearman’s rank test demonstrated the presence of very strong positive correlations between HP-^13^C-MRI-derived lactate SNR and RNAscope-derived total epithelial LDH density values extracted using the WMP-, T2WI-, and TC-SNR-guided segmentation approaches (ρ_s_ = 0.97, 0.96, 0.96, respectively; *p* < 0.0001; Fig. 3a–c). The same was observed for the correlation pair of HP-^13^C-MRI-derived *k*_PL_ and RNA-scope-derived epithelial *LDHA* density (ρ_s_ = 0.72, 0.75, 0.80 for WMP-, T2WI-, TCSNR-guided segmentations, respectively; *p* < 0.05; Fig. 3d–f).

## Discussion

This study compared HP-^13^C-MRI metabolic metrics in PCa derived from three segmentation approaches based on the histopathological gold standard of WMP, clinical ^1^H-MRI, and total carbon SNR maps. We show that despite significant differences in ROI surface areas and absolute values of the resulting metabolic metrics, all three segmentation approaches correlated strongly with the epithelial LDH mRNA expression that represents the tumour metabolic phenotype. These results provide quantitative evidence for the use of TC-SNR maps on HP-^13^C-MRI in addition to standard-of-care T2WI for reliably delineating PCa in the absence of the histopathological gold standard. Moreover, this study presents the rationale for using TC-SNR maps to target prostate biopsies to sample the most glycolytically active areas with the highest epithelial LDH expression.

To our knowledge, this is the first study to directly compare different HP-^13^C-MRI segmentation approaches in localised PCa. In their first-in-man study using HP-^13^C-MRI to image localised disease, Nelson et al [1] visually compared the locations of regions with high [1-^13^C]lactate labelling with abnormal areas on T2WI that contained biopsy-proven PCa foci, which is similar to the T2WI-guided approach utilised in this study. The same approach was used in a case report investigating androgen deprivation therapy–induced metabolic changes in a patient with biopsy-proven high-grade lesion [3]. In addition, two prospective studies in prostatectomy cohorts both utilised the WMP-guided approach that provides a definitive measure of tumour volume [2, 4]. Previously, we have used segmentation based on metabolism using preclinical and in silico models [11], whilst in this study we applied it to clinical data and validated it against tissue-based spatial transcriptomics for the first time. In this study, the TC-SNR approach involved setting up a specific SNR threshold aimed at removing potentially noisy data from HP-^13^C-MRI metabolic maps, which may not be reflective of true tissue metabolic properties. This can be particularly useful in cases with low polarisation, where the application of a TC-SNR threshold may also act as a quality control measure to determine the reliability of the signal produced. TC-SNR may also be helpful for demonstrating glycolytic activity in lesions which are ^1^H-MRI occult [1, 2, 4]. Thus, the application of a TC-SNR threshold may identify a significant focus of PCa and guide optimal sampling of the most glycolytically active tumour areas with targeted biopsy. In our study, the TC-SNR threshold was set at 5.0, which followed the strict Rose model decision strategy [9]. This was in line with real-life data where studies with median tumour-derived TC-SNR < 5.0 were considered a technical failure [4], and all areas of histopathologically proven cancer had TC-SNR > 5.0 [4, 6]. Importantly, the selected threshold allowed us to visualise areas with the highest combined epithelial LDH expression, suggesting the presence of a tumour “metabolic core” that produces the highest HP-^13^C-MRI signal and can be targeted using the TC-SNR approach. TC-SNR signal may also be heavily dependent on tumour perfusion, and therefore the vascular delivery of [1-^13^C]pyruvate. However, tumour vascular permeability to a gadolinium chelate is likely to be different from the perfusion of the smaller pyruvate molecule into the tumour, and TC-SNR may also be dependent on local magnetic field inhomogeneities. In support of this, we saw no significant correlations between *K*^trans^ and HP ^13^C-MRI parameters previously [4], but this comparison requires further investigation in larger cohorts.

The observed differences between WMP- and T2WI-guided ROI surface areas are in line with the known underestimation of tumour measurements on ^1^H-MRI [5]. However, the resulting HP-^13^C-MRI metrics were similar, which may be explained by a relatively low in-plane true resolution of HP-^13^C-MRI (12.5 × 12.5 mm^2^) coupled with a high spatial overlap between the two segmentation approaches, which was also reflected by the linear regression and Bland-Altman analyses. Therefore, T2WI alone is as reliable as the histopathological gold standard for HP-^13^C-MRI segmentation. Importantly, whilst TC-SNR-derived metabolic values were numerically different to those obtained using T2WI, the two segmentation approaches had strong linear relationship and agreement. In addition, lactate SNR measured using all three approaches showed strong positive correlations with the underlying tumour metabolic phenotype assessed by the epithelial mRNA density of LDH, the enzyme responsible for the imaged reaction [4]. This trend also held for *k*_PL_ and epithelial *LDHA*, which were shown to be closely related in a previous study [4]. Clinically, the use of TC-SNR-guided ROIs may be preferred due to the higher probability of sampling the metabolic core of the tumour that represents its most aggressive component [4].

This study has several limitations. The sample size was relatively small, albeit in line with similar publications in the field [1, 2, 6]. The impact of TC-SNR-guided segmentation on the ability of HP-^13^C-MRI to navigate targeted sampling of ^1^H-MRI occult lesions was not assessed since all patients already had biopsy-proven disease. The study excluded ^1^H-MRI occult lesions due to their inability to be prospectively segmented using the T2WI-guided segmentation approach.

In conclusion, the TC-SNR-guided segmentation approach generated smaller tumour regions with higher measures of metabolism in keeping with identification of the metabolic core on RNAscope. Although the T2WI approach generated larger regions with lower overall levels of metabolism, these were linearly correlated with the measures using the TC-SNR approach and those generated from the WMP gold standard. These findings support the use of TC-SNR maps for delineating localised PCa on HP-^13^C-MRI and targeting biopsies to sample the most glycolytically active tumour areas.

## Supplementary Information

ESM 1 (DOCX 2293 kb)

## Abbreviations

DSC: Dice similarity coefficient
HP-13C-MRI: Hyperpolarised [1-13C]pyruvate MRI
*k*_PL_: Pyruvate-to-lactate exchange rate constant
LDH: Lactate dehydrogenase
PCa: Prostate cancer
TC-SNR: Total carbon SNR
WMP: Whole-mount pathology

## Declarations

### Guarantor

The scientific guarantor of this publication is Dr. Tristan Barrett.

### Statistics and biometry

No complex statistical methods were necessary for this paper given the exploratory analyses used. The authors acknowledge the input of Dr. Oleg Blyuss of Queen Mary University of London who provided helpful guidance on the appropriate use of the Bland-Altman analysis.

### Study subjects or cohorts overlap

Some study subjects or cohorts have been previously reported in Sushentsev N, McLean MA, Warren AY, et al (2022) Hyperpolarised ^13^C-MRI identifies the emergence of a glycolytic cell population within intermediate-risk human prostate cancer. *Nat Commun* 2022 131 13:1–12. 10.1038/s41467-022-28069-2

### Methodology

• retrospective

• observational

• performed at one institution
