# Supplementary material for: The potential of hyperpolarised 13C-MRI to target glycolytic tumour core in prostate cancer
Source: Eur Radiol. 2022 Jun 22;32(10):7155–62. doi: 10.1007/s00330-022-08929-7 (PMC9474577; doi:10.1007/s00330-022-08929-7)
Supplement: Supplementary file 1 — (DOCX 2293 kb) [file 330_2022_8929_MOESM1_ESM.docx]

**The potential of hyperpolarised ^13^C-MRI to target glycolytic tumour core in prostate cancer**

**Supplementary Information**

| ***P*,  difference (T2WI-WMP)** | ***P*,**  **difference (TC-SNR-WMP)** | ***P*,**  **difference (TC-SNR-T2WI)** |
| --- | --- | --- |
| **Pyruvate SNR** | | |
| 0.60 | 0.54 | 0.004* |
| **Lactate SNR** | | |
| 0.164 | 0.05 | 0.26 |
| **TC-SNR** | | |
| 0.23 | 0.37 | 0.77 |
| ***k*_PL_** | | |
| <0.0001* | <0.0001* | <0.0001* |

**Supplementary Table 1.** Outcomes of the D’Agostino-Pearson test evaluating the normality of the distribution of differences in pyruvate SNR, lactate SNR, TC-SNR, and *k*_PL_ measured using different segmentation approaches. * denote differences that did not follow normal distribution.

**a**

**b**

**c**

**d**

**e**

**f**


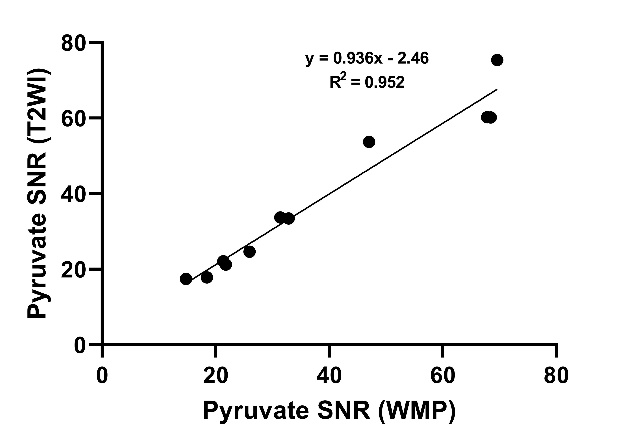

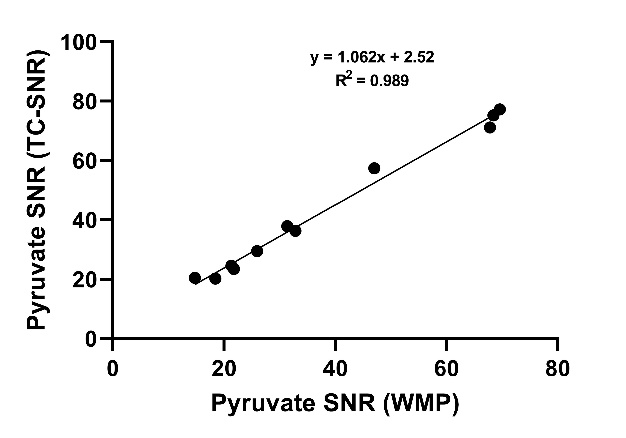

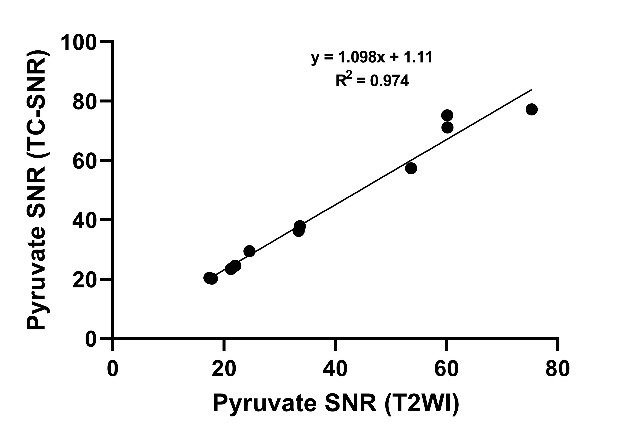

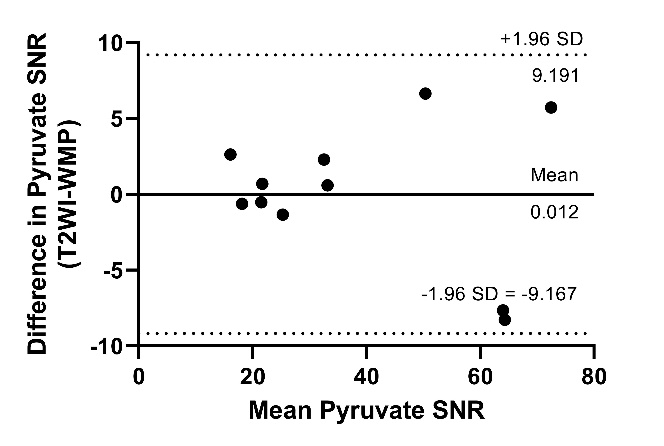

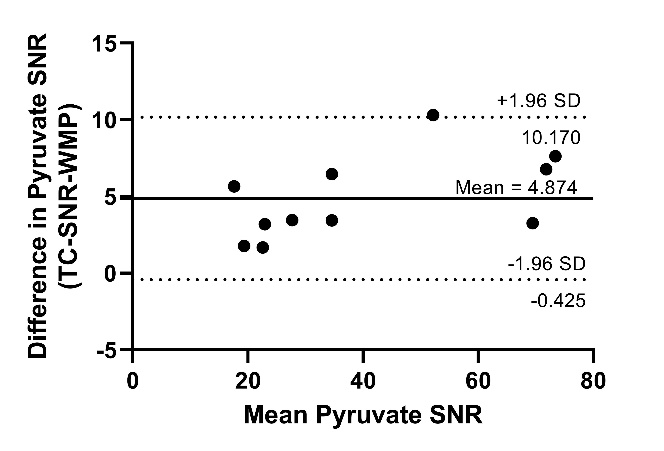

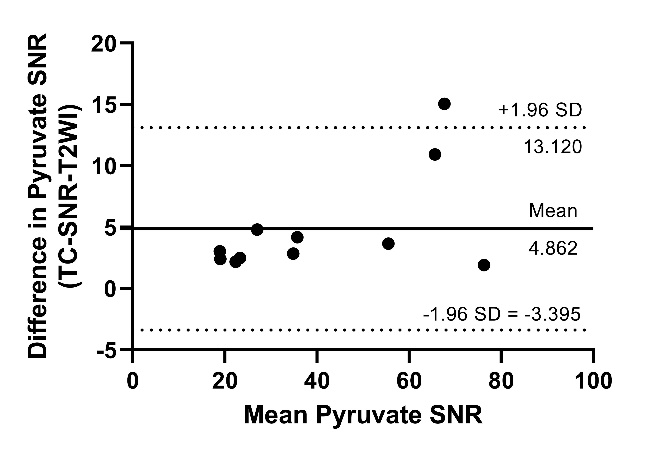


**Supplementary Figure 1.** Linear regression plots **(a, c, e)** and Bland-Altman plots **(b, d, f)** comparing HP ^13^C-MRI-derived pyruvate SNR values obtained using WMP-, T2WI-, and TC-SNR-guided segmentation approaches. **a, c, e** Linear regression plots include captions representing slopes of the linear fits, y-intercepts and coefficients of determination (R^2^). **b, d, f** Bland-Altman plots include dotted lines representing upper and lower 95% limits of agreement and bold lines representing the mean biases with appropriate captions included.

**a**

**b**

**c**

**d**

**e**

**f**


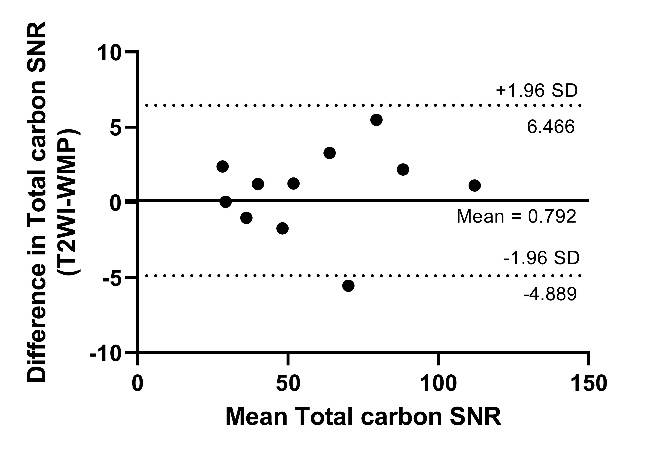

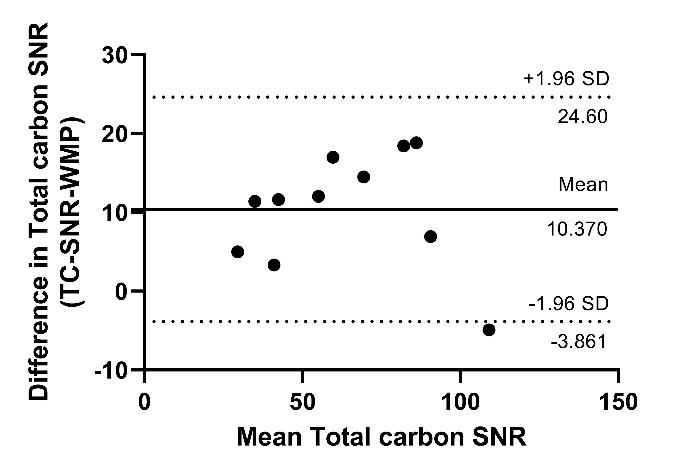

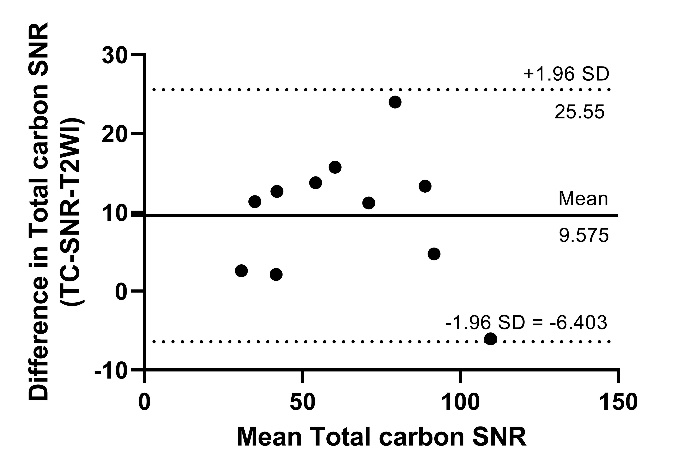

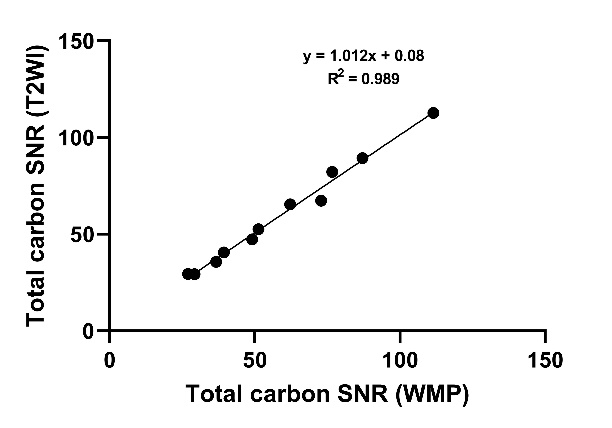

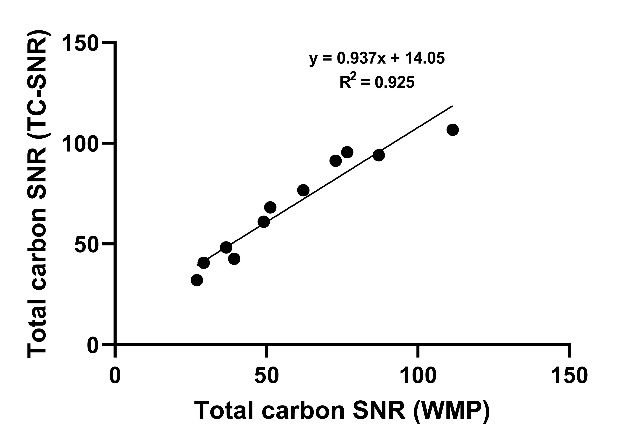

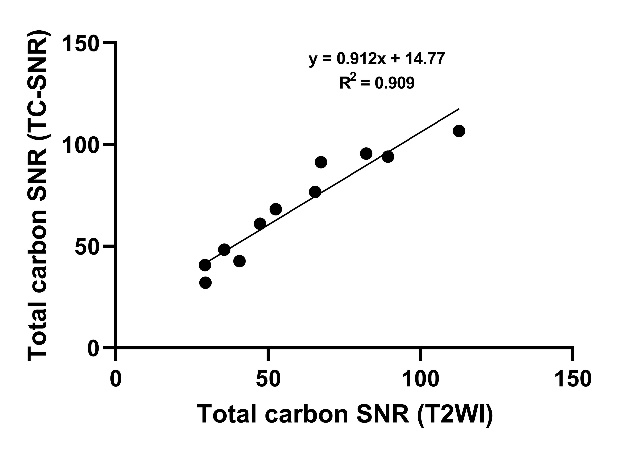


**Supplementary Figure 2.** Linear regression plots **(a, c, e)** and Bland-Altman plots **(b, d, f)** comparing HP ^13^C-MRI-derived total carbon SNR values obtained using WMP-, T2WI-, and TC-SNR-guided segmentation approaches. **a, c, e** Linear regression plots include captions representing slopes of the linear fits, y-intercepts and coefficients of determination (R^2^). **b, d, f** Bland-Altman plots include dotted lines representing upper and lower 95% limits of agreement and bold lines representing the mean biases with appropriate captions included.

**a**

**b**

**c**

**d**

**e**

**f**


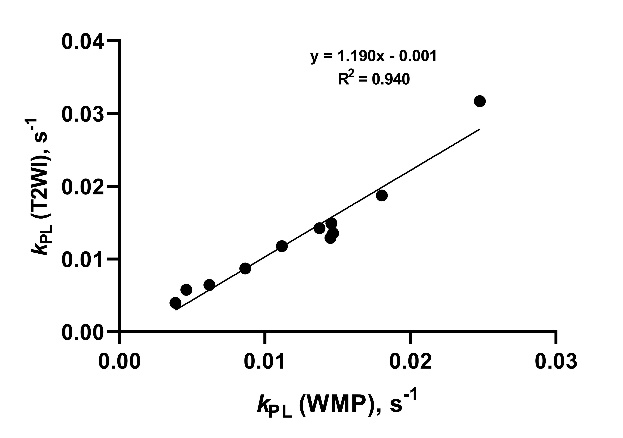

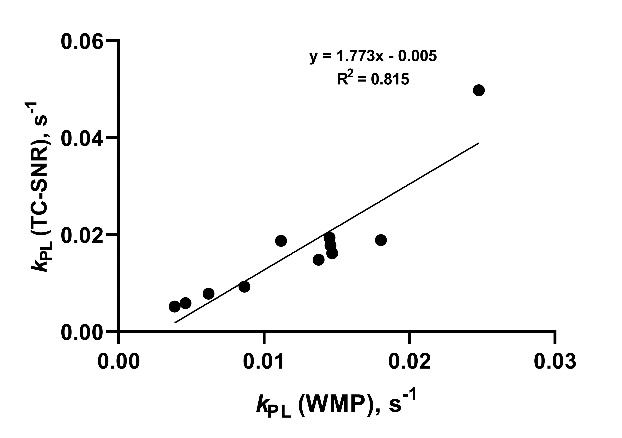

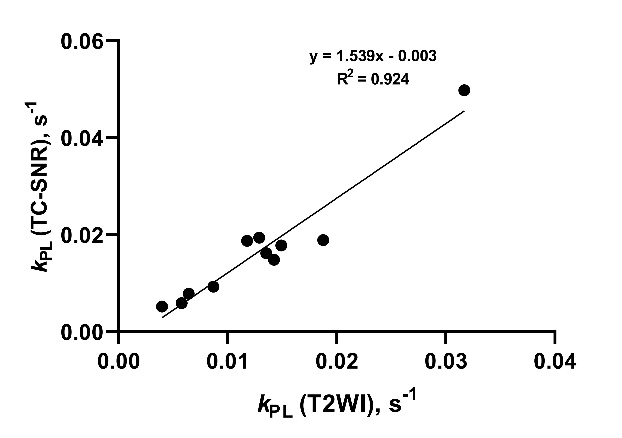

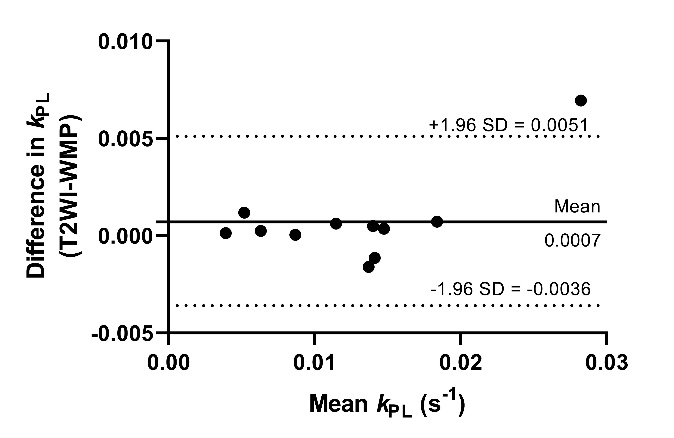

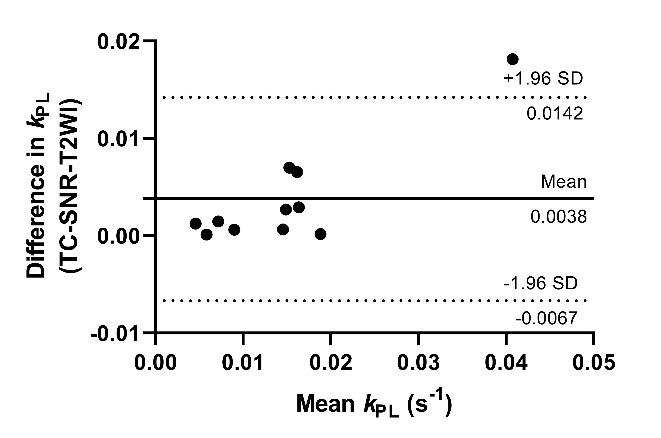

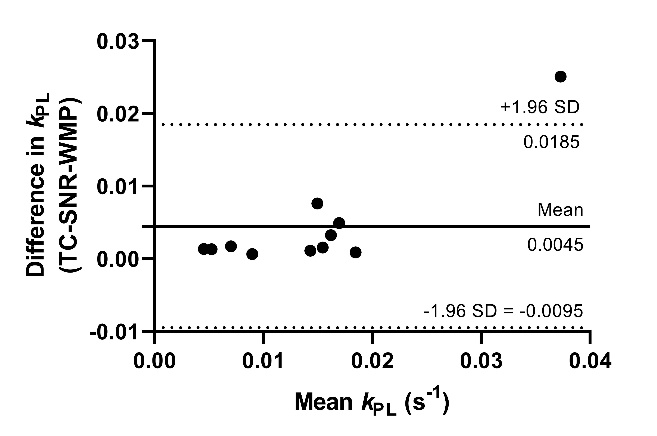


**Supplementary Figure 3.** Linear regression plots **(a, c, e)** and Bland-Altman plots **(b, d, f)** comparing HP ^13^C-MRI-derived *k*_PL_ values obtained using WMP-, T2WI-, and TC-SNR-guided segmentation approaches. **a, c, e** Linear regression plots include captions representing slopes of the linear fits, y-intercepts and coefficients of determination (R^2^). **b, d, f** Bland-Altman plots include dotted lines representing upper and lower 95% limits of agreement and bold lines representing the mean biases with appropriate captions included.
